# Preventive Effect of TU-100 on a Type-2 Model of Colitis in Mice: Possible Involvement of Enhancing Adrenomedullin in Intestinal Epithelial Cells

**DOI:** 10.1155/2013/384057

**Published:** 2013-11-19

**Authors:** Atsushi Kaneko, Toru Kono, Naoko Miura, Naoko Tsuchiya, Masahiro Yamamoto

**Affiliations:** ^1^Tsumura Research Laboratories, Tsumura & Co., 3586 Yoshiwara, Ami-machi, Inashiki-gun, Ibaraki 300-1192, Japan; ^2^Faculty of Pharmaceutical Sciences, Hokkaido University, Sapporo 060-0812, Japan; ^3^Center for Clinical and Biomedical Research, Sapporo Higashi Tokushukai Hospital, Sapporo 065-0033, Japan

## Abstract

*Purpose*. Crohn's disease (CD) and ulcerative colitis (UC), the two major forms of inflammatory bowel disease (IBD), have histopathologically and immunologically different characteristics. We previously reported that a traditional Japanese medicine, daikenchuto (TU-100), ameliorated a trinitrobenzenesulfonic acid- (TNBS-) induced type-1 model colitis exhibiting histopathological features of CD through adrenomedullin (ADM) enhancement. Our current aims were to examine whether TU-100 ameliorates a type-2 model colitis that histologically resembles UC and identify the active ingredients. *Methods*. TU-100 was administered orally to mice with oxazolone- (OXN-) induced type-2 model colitis. The morbidity was evaluated by body weight loss and the macroscopic score of colonic lesions. ADM was quantified using an EIA kit. *Results*. TU-100 prevented weight loss and colon ulceration. ADM production by intestinal epithelial cells was increased by TU-100 addition. Screening to identify active ingredients showed that [6]-shogaol and hydroxy **α**-sanshool enhanced ADM production. *Conclusions*. TU-100 exerted a protective effect in OXN-induced type-2 model colitis, indicating that TU-100 may be a beneficial agent for treatment of UC.

## 1. Introduction

Inflammatory bowel diseases (IBD), including Crohn's disease (CD) and ulcerative colitis (UC), are chronic progressive and destructive disorders of the gastrointestinal tract, characterized by inflammation associated with uncontrolled innate and adaptive immunity against normal bowel constituents like commensal bacteria and various microbial products [1]. Meanwhile, an etiologic role for splanchnic blood flow in IBD has been relatively neglected. The gastrointestinal epithelium is anatomically positioned to provide a selective barrier between the anaerobic lumen and lamina propria, which is supported by a complex vasculature. This important barrier is affected by reduced blood flow and resultant tissue hypoxia, particularly in sites of active inflammation in individuals with IBD [2]. Daikenchuto (TU-100), a pharmaceutical-grade traditional Japanese (*kampo*) medicine, has been widely used for the treatment of various gastrointestinal disorders including postoperative ileus [3]. TU-100 has been integrated into the modern medical care system in Japan as a prescription drug. We recently clarified the mechanism by which TU-100 increases intestinal blood flow [4]. TU-100 activates transient receptor potential ankyrin 1 (TRPA1) expressed on intestinal epithelial cells, followed by release of the vasodilator peptide adrenomedullin (ADM). The vasodilatory effect of TU-100 has been shown in clinical [5] and experimental studies [4, 6–9]. ADM was initially identified as a vasodilator and is thought to be the most potent endogenous vasodilatory peptide found in the body [10, 11]. Other effects of ADM include augmentation of the tolerance of cells to oxidative stress and hypoxic injury, angiogenesis, anti-inflammation, and antibiotic activity. Moreover, several reports indicate that administration of ADM prevented development of colitis in experimental IBD models [12–18].

CD and UC have immunologically different pathobiologies. The responding T cells in CD and UC exhibit T helper cell (Th) 1 and 2 phenotypes, respectively. Among various experimental colitis models, hapten-induced colitis in rodents caused by intrarectal administration of trinitrobenzene sulfonic acid (TNBS) and oxazolone (OXN) is thought of as a type-1 colitis animal model resembling CD and a type-2 model resembling UC, respectively. Therefore, therapeutic strategies for IBD include the use and development of various immunomodulating agents as the most powerful and promising methods.

We previously reported that TU-100 ameliorated TNBS-induced type-1 colitis in mice via enhancement of intestinal release of ADM [19]. Considering that the effect of TU-100 is related to endogenous ADM in the intestines, we hypothesized that TU-100 would be effective in treatment of other types of colitis with different immunological properties. The aim of the present study was to clarify whether TU-100 has a beneficial effect on an OXN-induced type-2 model colitis.

## 2. Materials and Methods

### 2.1. Test Samples

Daikenchuto extract in the form of a dried powder was obtained from Tsumura & Co. (Tokyo, Japan), which manufactures it as an aqueous extract containing processed ginger, ginseng radix, and Japanese pepper in the ratio of 5 : 3 : 2. The extract yielded 12.5% by weight. TU-100 was prepared by mixing daikenchuto extract powder and maltose syrup powder (Tsumura & Co.) at a ratio of 1 : 8. Hydroxy **α**-sanshool was extracted from Japanese pepper at Tsumura & Co. with a purity of 97.9%. [6]-Gingerol, [6]-shogaol, ginsenoside Rb1, ginsenoside Rg1, ginsenoside Rd, and maltose were purchased from Wako Pure Chemical Industries, Ltd. (Osaka, Japan). Xanthoxylin was purchased from Tokyo Chemical Industry (Tokyo, Japan). 

### 2.2. Animals

Five-week-old male C57BL/6CrSlc mice were purchased from Japan SLC (Shizuoka, Japan). The animals were allowed free access to water and standard laboratory food and housed at a temperature of 23 ± 2°C, relative humidity of 55 ± 10%, and a 12 h light : 12 h dark cycle, with lights on from 07:00 to 19:00 h daily. All experimental procedures were performed according to the “Guidelines for the care and use of laboratory animals” of Tsumura & Co. Ethical approval of the experimental procedures used in this study was obtained from the Laboratory Animal Committee of Tsumura & Co. 

### 2.3. OXN-Induced Colitis

Colitis was induced following the methods described by Hyun et al. [20] with slight modification. Briefly, under pentobarbital anesthesia, 100 **μ**L of 1% OXN (4-ethoxymethylene-2-phenyl-2-oxazolin-5-one, Sigma-Aldrich, St. Louis, MO) dissolved in a mixture of four parts acetone to one part olive oil was applied to the shaved abdominal skin of mice. One week later, 100 **μ**L of 50% ethanol solution with or without 1% OXN was instilled rectally under anesthesia with pentobarbital and atropine (Sigma-Aldrich, 3 mg/kg, i.p.). The mice were held in a vertical position (head down) for 30 seconds and then put back into their cages. After 4 d, the mice were sacrificed, the colon was dissected, and macroscopic colonic lesions were graded on a scale from 0 to 11 based on criteria reflecting hemorrhage (0-1), edema (0-1), stricture (0-1), ulceration (0-1), fecal blood (0-1), mucus (0-1), diarrhea (0-1), erythema (0, absent; 1, less than 1 cm; and 2, more than 1 cm), and adhesion (0, absent; 1, moderate; and 2, severe). This animal test rarely causes death, but one mouse among 16 colitis control mice died during this experiment. We evaluated the disease activity score excluding the dead mouse.

TU-100 in distilled water was given orally at 900 mg/kg to colitis mice several hours, 1, 2, and 3 d after OXN instillation. The dose of TU-100 given to mice was based on previous publications on experimental TU-100 studies, which reported the beneficial effects of TU-100 related to clinical efficacy. Further, the dose used in the present study was expected to produce blood concentrations of the major ingredients in mice similar to those in humans (data not shown). 

### 2.4. ADM Production Test

Rat small intestine epithelial cell lines IEC-6 and IEC-18 were obtained from DS Pharmaceuticals (Osaka, Japan) and grown in DMEM supplemented with 10% heat-inactivated fetal bovine serum (FBS), 2 mmol/L L-glutamine, 100 U/mL penicillin, 100 **μ**g/mL streptomycin, and 10 mmol/L HEPES. IEC-6 or IEC-18 cells between the 30th and 37th passage were plated in 96-well flat-bottom microtiter plates at 1 × 10^4^ cells/well in DMEM supplemented with the same additives described above, allowed to settle overnight, and then culture fluids were replaced with fresh medium containing 3% FBS and test sample. TU-100 was added to cultures at final concentrations of 270, 900, and 2700 **μ**g/mL after being passed through a 0.45 **μ**m filter. Cells were incubated for 24 h, and ADM in the culture fluids was quantified using an enzyme immunoassay kit specific for rat ADM according to the instructions provided by the manufacturer (Phoenix Pharmaceuticals, Burlingame, CA). The lowest level of detection for ADM was 10 pg/mL.

### 2.5. Cell Growth Test

IEC-6 cells were treated with the test sample as described above. After culture fluids were removed for ADM quantification, cell growth activities were measured using an XTT reduction assay kit (Biological Industries, Beit Haemek, Israel) under the manufacturer's instructions. Optical density at 465 nm was measured by subtracting the reference absorbance at 630 nm. The optical density of the medium-only wells was in a range of 0.07 to 0.10.

### 2.6. Cytokine Induction Tests

Spleen mononuclear cells were isolated from normal C57BL/6CrSlc mice. Erythrocytes were removed from a spleen cell suspension by hypotonic lysis in ammonium chloride and potassium chloride buffer. The cells were seeded in 96-well flat-bottom microtiter plates (5 × 10^5^ cells/mL) in RPMI 1640 medium supplemented with 10% FBS, 100 units/mL penicillin and 100 **μ**g/mL streptomycin, 10 mmol/L HEPES, and 50 **μ**mol/L 2-mercaptoethanol and stimulated with *E. coli*-derived lipopolysaccharide (LPS, 1 **μ**g/mL, Sigma-Aldrich) for 2 d or an antibody against mouse CD3 (anti-CD3, clone 145-2C11, 1 **μ**g/mL; BD Biosciences, San Jose, CA) for 1 d. ADM was added to cultures at 0.01, 0.1, or 1 **μ**mol/L. Culture supernatants were collected and then stored at −80°C until the cytokine assay. Cytokines were measured using a Bio-Plex Pro mouse cytokine assay panel (Bio-Rad Laboratories, Inc., Hercules, CA) according to the manufacturer's instructions. IL-17A and TNF-**α** were measured using conventional ELISA assay reagents produced by BD Biosciences and R&D Biosystems (Minneapolis, MN), respectively. The lowest levels of detection were 9.36 (IL-1**β**), 0.86 (IL-2), 0.28 (IL-4), 2.77 (IL-5), 0.21 (IL-6), 0.87(IL-10), 5.06 (IL-12p70), 11.55 (IL-13), 11.09 (GM-CSF), 15.54 (IFN-**γ**), 37.14 (MCP-1), and 4.11 (IL-17A and TNF-**α**).

### 2.7. Statistical Analysis

All values are expressed as the mean ± SEM. Statistical significance was evaluated by one- or two-way analysis of variance (ANOVA), and a probability of less than 0.05 was considered significant at Student's *t*-test or Dunnett's test. 

## 3. Results

### 3.1. Ameliorating Effect of TU-100 in OXN-Induced Colitis

**Figure 1:**
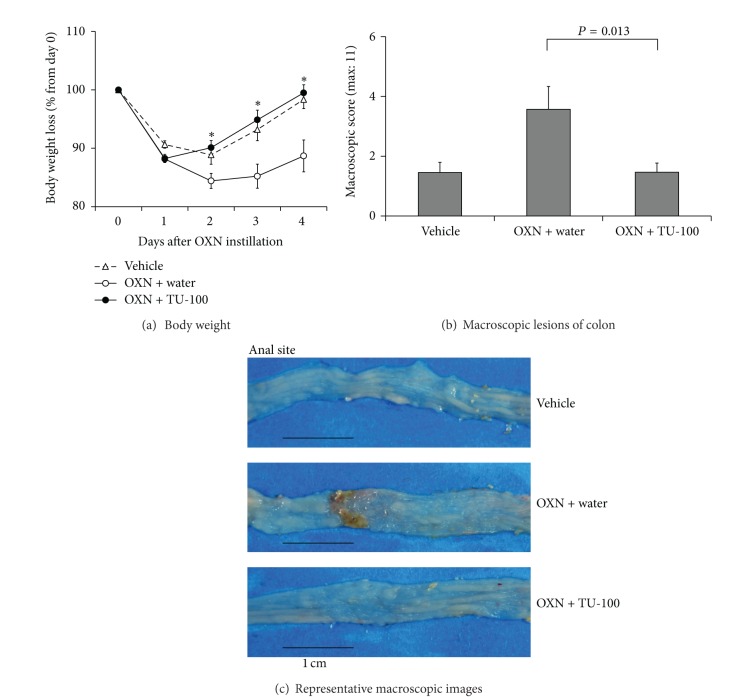
Effects of TU-100 on body weight and macroscopic colon lesions in OXN-induced colitis. Mice were presensitized with oxazolone (OXN, 1 mg/100 **μ**L applied to skin), and then OXN (1 mg/100 **μ**L) or vehicle, 50% ethanol, was instilled intrarectally (i.r.). TU-100 (900 mg/kg) was given orally to mice several hours, 1, 2, and 3 days after OXN instillation, and mice were sacrificed 4 days after OXN instillation. Body weight changes after OXN instillation (a), and macroscopic scores of colon lesions (b) are shown. Representative macroscopic images of each group are shown (c). Vehicle: 50% ethanol i.r. + water p.o., *N* = 11, OXN + water: OXN i.r. + water p.o., *N* = 14, OXN + TU-100: OXN i.r. + TU-100 p.o., *N* = 15, *P < 0.05 versus colitis control by Student's *t-*test.

The OXN-treated mice developed rapid-onset colitis marked by weight loss, diarrhea, and bloody stool. The body weight of the OXN-treated group decreased transiently from day 1 to day 2 after OXN instillation. Oral administration of TU-100 at 900 mg/kg, the dosage for treatment of the TNBS-induced colitis model [19], resulted in a marked prevention of ulcerative colitis, as well as a reduction in the loss of body weight (Figure 1).

### 3.2. ADM Enhancement by TU-100 Addition

**Figure 2:**
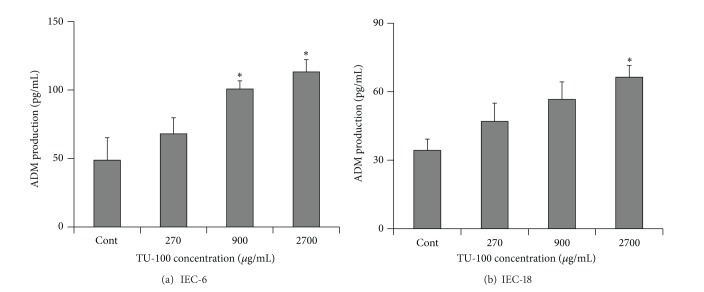
Adrenomedullin-enhancing activity of TU-100 in intestinal epithelial cells. Intestinal epithelial cell lines IEC-6 (a) and IEC-18 (b) settled overnight were incubated with the indicated concentrations of TU-100 for 1 day. Adrenomedullin (ADM) in the culture fluid was determined by ADM-specific EIA. *N* = 4 (a), 3 (b). *P < 0.05 versus control by Dunnett test.

Next, we investigated the effect of TU-100 on cultures of IEC-6 and IEC-18 cells; the former were used in our previous studies [4, 7, 19], and Kishikawa et al. used the latter for demonstration of LPS-induced ADM synthesis in intestinal epithelial cells [21]. As shown in Figure 2, TU-100 increased ADM production by both IEC-6 cells and IEC-18 cells in a concentration-dependent matter.  Table 1 shows the results of screening the major ingredients of TU-100 for their stimulatory effect on ADM in IEC-6. [6]-Shogaol, an ingredient of processed ginger, and hydroxy *α*-sanshool, an ingredient of Japanese pepper, have been shown to stimulate ADM from IEC-6 cells. Neither active ingredient showed a significant inhibition on cell growth following their additions. Essentially the same results were obtained for the experiment using IEC-18 cells (data not shown). 

### 3.3. Inhibition of Proinflammatory Cytokines by ADM Addition

ADM is known to have suppressive effects on proinflammatory cytokines such as TNF-**α** in various inflammation models [15]. Therefore, we examined the effects of ADM on cytokine production by murine spleen cells (Table 2). In the anti-CD3 stimulation assay, ADM significantly suppressed the production of IL-13, GM-CSF, IFN**γ**, and TNF-**α**, but not IL-1**β**, IL-2, IL-4, IL-5, IL-6, IL-10, IL-12p70, or IL-17A, while ADM augmented IL-2 production only at the highest concentration. Furthermore, ADM inhibited the LPS-induced production of GM-CSF, IFN**γ**, MCP-1, and TNF-**α**, but not IL-2, IL-6, IL-10, or IL-12p70. These data show that ADM is not an immunomodulator that deflects either the Th1 or Th2 response as far as the profile of cytokine productions was examined.

## 4. Discussion

We examined the large intestines of TU-100-treated mice by macroscopic and histological observations, but found no change compared with those of nontreated mice (data not shown). The histological features of the colon in OXN-treated mice were as previously reported [20]. Superficial inflammation was characterized by epithelial cell loss and/or regenerative epithelium, depletion of goblet cells, inflammatory cell infiltration composed mainly of neutrophils and eosinophils, edema formation, hemorrhage, vascular dilatations, and occasionally crypt abscesses. In the present study, we observed that OXN-treated mice exhibited typical characteristics of OXN-induced colitis such as hemorrhage, edema, ulceration, diarrhea, and erythema. Compared with the colitis control group, mice in the TU-100-treated colitis group exhibited remarkable alleviation of these colitis processes.

CD and UC have traditionally been distinguished by patterns of helper T-cell dysfunction. Lamina propria cells from patients with CD overproduce cytokines associated with a Th1 response, such as IL-12 and IFN-**γ**. In contrast, cells from patients with UC overproduce cytokines associated with the Th2 response, such as IL-5 and IL-13. Studies of mouse models of mucosal inflammation (e.g., type-1 TNBS- and type-2 OXN-induced colitis) have provided further evidence that activities of Th1 and Th2 cells mediate the pathogenesis of CD and UC, respectively [1]. Therefore, the effects of immunomodulating agents on CD, UC, and their disease models have been shown to be different, sometimes even contradictory. For example, a certain anti-TNF monoclonal antibody has been reported to be effective in TNBS-induced colitis but not in OXN-induced colitis [22]. Further, infection with Helminth parasites ameliorates TNBS-induced colitis but exaggerates OXN-induced colitis via eosinophilia and elevated IL-5 [23].

However, distinguishing CD from UC based on overproduction of Th1 and Th2 cytokines is an oversimplification of a complex immunological response. Much of the inflammatory pathology originally believed to be mediated by Th1 cells and IL-12 has been found to be mediated by a subset of T cells, Th17 cells, which produce the IL-17 family members IL-21 and IL-22 at sites of inflammation and require the IL-12 family member IL-23 as a growth factor [24]. Adding to the complexity, Th1, Th2, and Th17 cells have both pro- and anti-inflammatory properties in various types of mucosal inflammation [25].

Further, there are commonalities underpinning their pathogenesis. The pathogenesis of IBD includes a complex interaction between innate and adaptive immune cells, local immune modulators and cytokines, intestinal vasculature, nutritional factors, and enteric microbiota. Therefore, intervention by dietary management and probiotic ingestion are still clinically effective therapeutic options for CD and UC [26–28], though they have yet to attain an acceptable evidence-based status. In addition, a variety of agents such as FK506-entrapped nanoparticles [29], viral and nonviral NF-*κ*B decoys [30, 31], blockade of CD30-CD30L interaction [32], IL-25 [33], leptin [34], and a plant-derived compound [35] have been shown to ameliorate both colitis models. TU-100 showed ameliorating effects on TNBS-induced colitis in previous studies [7, 19] and OXN-induced colitis in the present study. TU-100 is, therefore, a candidate for a novel therapeutic agent for both CD and UC. Though the clinical efficacy of TU-100 in IBD is still unclear, accumulating case reports in Japan suggest the possible efficacy of TU-100 in a wide variety of gastrointestinal disorders including CD and UC. Further, placebo-controlled double blind studies of TU-100 on IBD are now in progress in the US (NCT01388933).

Although the mechanism by which TU-100 exerts its ameliorating effects on OXN-induced colitis remains to be elucidated, it is plausible that the ADM released by TU-100 stimulation plays several roles. As we previously reported, intestinal epithelial cells of the small and large intestines produce much ADM and release it following stimulation of TRPA1 channels expressed on their plasma membranes [4, 7, 19]. There was no difference between the small and large intestines in ADM expression and responses to TU-100 stimulation followed by ADM production. Hydroxy **α**-sanshool and [6]-shogaol are known to be nonselective agonists of TRPA1 channels [36]. Therefore, exposure of intestinal epithelial cells to them results in increases of ADM production and release. 

The representative Th2 cytokine IL-13 is expressed abundantly in the inflamed intestinal mucosa and reported to be profoundly involved in development of OXN-induced colitis [37]. Our studies examining the regulatory effects of ADM on cytokine induction showed that ADM inhibited IL-13 produced by anti-CD3 stimulation. Overall, ADM has demonstrated anticolitis effects in TNBS- [15, 18], DSS- [12, 16, 17], and acetic-acid-induced [14] colitis models. The suppressant effect of TU-100 on mucosal damage [19], mucosal ischemia [7], and especially TNF-**α** production was shown in TNBS-induced colitis in an ADM-dependent manner. It should be noted that ADM potently decreased TNF-**α** production induced by both anti-CD3 and LPS in the present study. It is reported that ADM upregulates intracellular cAMP, which activates protein kinase A and CREB pathways, resulting in suppression of transcriptional factors, such as NF-kB, that are critical for induction of proinflammatory cytokines [38]. TNF-**α** has been known to be an extraordinarily important pathogenic factor in CD and UC, and an anti-TNF-**α** antibody is an effective treatment option for patients with moderate to severe UC with an inadequate response to conventional glucocorticoid treatment [39] as well as CD patients. Only IL-2 production by CD3-stimulated cells was significantly enhanced by addition of 1 **μ**mol/L ADM. IL-2 is a pleiotropic cytokine produced by naïve T cells and activated Th1 cells and was identified originally as a growth factor for various lymphocytes [40]. T cells increase the beta subunit CD122 of IL-2 receptors following activation to form a high affinity IL-2 receptor with alpha subunit CD25. ADM evidently suppressed T cell activation as far as focusing on the other cytokines downregulation and may decrease consumption of native T cell-derived IL-2. 

In considering the anticolitis effect of TU-100 through enhancement of ADM production, not only the anti-immune action but also the multifunctional effects of ADM should be noted.Our previous study showed that TU-100 improved microvascular circulation at inflammatory ischemia sites of colitis via endogenous ADM release [7]. ADM is reported to protect against inflammatory hypoxia-induced damage of the intestinal epithelium through fine tuning of hypoxia-induced factor stabilization in a DSS-induced colitis model [17]. Moreover, ADM enhanced expression of the epithelial intercellular junctions such as tight and adherence junctions, followed by improvement of hyperactivation and hyperpermeability of the intestinal epithelium in a DSS-induced colitis model [12]. Antimicrobial effects of ADM are known [41] and may affect the development of colitis. 

Considering the results shown in Table 2, [6]-shogaol and hydroxy **α**-sanshool are suggested to be the major active ingredients of TU-100 for stimulation of ADM release and presumably the main ingredients of TU-100 that express anticolitis effects. Ginger-derived various ingredients like gingerols and shogaols are known to inhibit cyclooxygenase enzymatic activity and expression and block intracellular signals induced by inflammatory factors [42, 43]. In addition, ginseng-derived ginsenosides are also reported to exhibit various biological effects like anti-inflammation and antiapoptosis [44]. TU-100, therefore, has many active ingredients and should be considered a multitarget agent, although we focused on ingredients to increase ADM in this study.

## 5. Conclusions 

We showed that TU-100 prevented development of an OXN-induced type-2 model colitis. TU-100-induced ADM may be involved in the mechanism of the ameliorative effects. TU-100 may open the way to the development of novel preventive strategies for IBD, especially as a new agent to delay acute aggravation and maintain remission in UC patients.

## Figures and Tables

**Table 1:** Screening for ingredients that enhance ADM production.

Test sample origin	Ingredient	Concentration (*μ*mol/L)	Growth activity (optical density)	ADM production (pg/mL)
—	Control	—	1.419 ± 0.045	85.3 ± 4.9

Processed ginger	[6]-Gingerol	3	1.429 ± 0.016	99.7 ± 12.4
		30	1.400 ± 0.025	93.0 ± 6.5
	[6]-Shogaol	3	1.504 ± 0.019	100.0 ± 9.3
		30	1.363 ± 0.023	246.3 ± 5.0*

Ginseng radix	Ginsenoside Rb1	3	1.399 ± 0.014	98.0 ± 2.6
		30	1.366 ± 0.047	90.7 ± 9.2
	Ginsenoside Rg1	3	1.393 ± 0.067	89.0 ± 5.1
		30	1.375 ± 0.040	95.7 ± 3.8
	Ginsenoside Rd	3	1.379 ± 0.045	88.3 ± 5.4
		30	1.386 ± 0.026	103.3 ± 12.3

Japanese pepper	Xanthoxylin	3	1.429 ± 0.030	89.3 ± 7.4
		30	1.395 ± 0.012	99.0 ± 12.1
	Hydroxy *α*-sanshool	3	1.449 ± 0.009	110.3 ± 12.5
		30	1.557 ± 0.013*	120.3 ± 8.8*
		100	1.493 ± 0.043	162.0 ± 1.7*

Maltose syrup	Maltose	30	1.484 ± 0.019	106.7 ± 11.0
		300	1.467 ± 0.031	106.7 ± 11.7

IEC-6 cells were settled overnight and cultured for 1 d with or without the test sample at the indicated concentrations. ADM in the culture fluids was measured by EIA. Cell growth was measured using an XTT reduction assay kit. *N* = 3. *P < 0.05 versus control by Dunnett test.

**Table:** (a) Anti-CD3 stimulation

Cytokine	ADM (*µ*mol/L)			
	Control	0.01	0.1	1
IL-1*β*	29 ± 5	24 ± 3	24 ± 3	33 ± 9
IL-2	448 ± 37	460 ± 27	475 ± 31	595 ± 28*
IL-4	54 ± 3	50 ± 2	47 ± 3	50 ± 1
IL-5	2 ± 0	4 ± 1	2 ± 0	3 ± 1
IL-6	91 ± 4	94 ± 8	94 ± 4	109 ± 4
IL-10	12 ± 1	14 ± 0	11 ± 1	12 ± 1
IL-12p70	20 ± 3	17 ± 4	19 ± 3	19 ± 5
IL-13	140 ± 4	126 ± 12	90 ± 7*	91 ± 3*
IL-17A	103 ± 2	102 ± 6	106 ± 12	102 ± 12
GM-CSF	81 ± 5	75 ± 1	57 ± 2*	54 ± 4*
IFN-*γ*	3,821 ± 161	3,177 ± 313	2,110 ± 39*	1,959 ± 117*
TNF-*α*	16 ± 0	14 ± 0	10 ± 1*	8 ± 1*

**Table:** (b) LPS stimulation

Cytokine	ADM (*µ*mol/L)			
	Control	0.01	0.1	1
IL-6	326 ± 9	338 ± 4	350 ± 17	328 ± 13
IL-10	551 ± 50	551 ± 10	523 ± 30	472 ± 12
IL-12p70	22 ± 5	15 ± 5	10 ± 3	16 ± 3
GM-CSF	31 ± 3	27 ± 3	19 ± 2*	18 ± 2*
IFN-*γ*	44 ± 15	36 ± 12	N.D.	N.D.
MCP-1	262 ± 15	260 ± 24	161 ± 12*	149 ± 28*
TNF-*α*	81 ± 3	72 ± 4	37 ± 3*	28 ± 2*

Murine spleen cells were stimulated with 1 *μ*g/mL anti-CD3 for 1 d (a) or 1 *μ*g/mL LPS for 2 d (b). Adrenomedullin (ADM) was added to culture medium at the indicated concentrations. Cytokines were measured using a Bio-Plex mouse cytokine multiplex kit. Measurements of IL-17A and TNF-*α* were performed by conventional ELISA. Cytokine productions with no stimulus were 9.9 ± 1.6 in IL-2, 1.6 ± 0.2 in IL-6, 1.7 ± 0.2 in IL-10, and below the detection limit for the others. *N* = 3-4. *P < 0.05 versus control by Dunnett test.
